# Dr. Norman K. MacLeod

**Published:** 1913-05

**Authors:** 


Dr. Norman K. MacLeod, Toronto, 1903, died at his residence, 148 Delaware avenue, Buffalo, April, of pneumonia, having been ill two weeks. He was born in Brantford, Ont., 34 years ago, went to Melbourne, Australia, when a boy, and graduated from Melbourne University. Returning to Canada, he studied medicine at the University of Toronto, and after graduating in medicine went to London for post graduate work, studying under Sir A. E. Wright and others and devoting himself especially to research in regard to bacterial vaccines, though broadly interested in pathology and applying his special skill along practical lines. He joined his brother, Dr. James MacLeod, in Buffalo in 1907, and soon became well known, not only for his scientific research and by his professional acquaintance, but for his general scholarship and his charming character. In the best sense of the words, he was socially active and deservedly popular, though a hard worker in his laboratory and having the rare ability to combine technical science with clinical acumen.

